# Identification of Chromone Hybrids as Interleukin‐6 and Acetylcholinesterase Inhibitor for treatment of Alzheimer’s Disease

**DOI:** 10.1002/alz.089026

**Published:** 2025-01-09

**Authors:** Sukhvir Kaur, Yogita Bansal

**Affiliations:** ^1^ Chitkara University School of Pharmacy, Chitkara University, Himachal‐Pradesh, India, Baddi India; ^2^ Department of pharmaceutical sciences and drug research, Punjabi University, Patiala, Patiala India; ^3^ Department of Pharmaceutical Sciences and Drug Research, Punjabi University, Patiala, Patiala India

## Abstract

**Background:**

Neuroinflammation plays an important role in progression of Alzheimer’s disease (AD). Interlukin‐6 (IL‐6) is well identified marker in initiating and regulating inflammation, and formation of senile plaques in brain. Therefore, simultaneous inhibition of both IL‐6 and acetylcholinesterase (AChE) may be an effective strategy for AD.

**Method:**

Four series of molecules are designed by coupling a chromone, and a N, N‐disubstituted amine as pharmacophore for IL‐6 and AChE inhibition, respectively through an alkyl linker of different lengths (1‐4 carbon atoms). Designed compounds were docked into AChE to identify the best fit compounds for calculating ADME, synthesis and evaluation of AChE inhibitory activity. The compounds showing >45% inhibition of *Ee*AChE are selected for evaluation of IL‐6, AChE and butyrylcholinesterase (BuChE) inhibitory activities. The compound found to be most potent against was finally evaluated *in vivo* using STZ‐induced amnesia model in mice at three doses (0.2, 0.4 and 0.8 mg/kg).

**Result:**

Docking of all designed compounds in AChE pocket identify the 16 best fit compounds (Docking score > 8.3). The data suggests that a 2‐ or 3‐carbon atom linker is the most conducive to orient the pharmacophore for optimum binding with AChE active site. The predicted ADME properties of the 16 selected compounds suggest that these can cross the blood brain barrier (BBB) with good oral bioavailability. These compounds are synthesised and evaluated for anti‐AChE activity. Six compounds, showing >45% inhibition of AChE, are further evaluated for AChE, BuChE and IL‐6 inhibitory activities. Compound YS3g is found to be the most potent inhibitor of *Ee*AChE (IC_50_ 0.54 μM/ml) as well as IL‐6 (IC_50_ 0.57 μM/ml). YS3g was evaluated *in vivo* at three different doses (0.2, 0.4 and 0.8 mg/kg) and show dose‐dependent effects. At higher dose (0.8 mg/kg), it reverses the STZ‐induced memory deficit *in vivo* and shows histopathology similar to that shown by normal‐control animal.

**Conclusion:**

The results implied hybrid compounds formed by combing chromone with piperazine ring through a three‐carbon atom chain might act as promising small‐molecule agent for further investigation against AD.

